# MiR-23b targets cyclin G1 and suppresses ovarian cancer tumorigenesis and progression

**DOI:** 10.1186/s13046-016-0307-1

**Published:** 2016-02-13

**Authors:** Jing Yan, Jing-yi Jiang, Xiao-Na Meng, Yin-Ling Xiu, Zhi-Hong Zong

**Affiliations:** Department of Biochemistry and Molecular Biology, College of Basic Medicine, China Medical University, 100013 Shenyang, China; Department of Gynecology, The First Affiliated Hospital of China Medical University, 110001 Shenyang, China

**Keywords:** miR-23b, CCNG1, Ovarian cancer, Tumorigenesis, Progression

## Abstract

**Background:**

It has been proposed that cyclin G1 (CCNG1) participates in p53-dependent G_1_–S and G_2_ checkpoints and might function as an oncogenic protein in the initiation and metastasis of ovarian carcinoma. MicroRNA 23b (miR-23b) is a critical regulatory factor in the progression of many cancer cell types that targets the relevant genes.

**Methods:**

MiR-23b expression in ovarian tissues was quantified by quantitative reverse transcription–PCR. The ovarian cancer cell lines OVCAR3, HO8910-PM, and SKOV3/DDP were transfected with miR-23b, after we assayed the cell phenotype and expression of the relevant molecules. Dual-luciferase reporter assay and a xenograft mouse model were used to examine the expression of miR-23b and its target gene *CCNG1*.

**Results:**

*MIR23B* mRNA expression was significantly lower in epithelial ovarian carcinoma and borderline tumors than in normal ovarian tissues and benign tumors, and miR-23b expression among ages and pathological subtypes was significantly different. *CCNG1* mRNA expression was significantly lower in normal ovarian tissues than in benign tumors, borderline tumors, and ovarian carcinomas, and expression among pathological subtypes was significantly different. MiR-23b overexpression inhibited ovarian cancer cell proliferation, invasion, and migration, and induced apoptosis. Dual-luciferase reporter assay showed that miR-23b bound with the 3′ untranslated region of *CCNG1*. MiR-23b overexpression significantly downregulated CCNG1, urokinase, survivin, Bcl-xL, P70S6K, and matrix metallopeptidase-9 (MMP9) mRNA and protein expression. Furthermore, miR-23b inhibited tumor growth and suppressed CCNG1 expression in vitro.

**Conclusions:**

Our findings show that miR-23b may inhibit ovarian cancer tumorigenesis and progression by downregulating CCNG1 and the expression of the relevant genes. MiR-23b is a potentially novel application for regulating ovarian carcinoma progression.

## Background

The incidence of ovarian cancer is perpetually high: 200,000 new cases are diagnosed annually worldwide. Each year, it constitutes 4 % of all cancers diagnosed in women, and there are 6.6 new cases per 100,000 women per year [1, 2]. Globally, ovarian cancer caused 160,500 deaths in 2010, an increase from the 113,600 in 1990 and the 140,200 in 2008 [3, 4]. It also remains the most lethal gynecologic malignancy owing to late detection, intrinsic and acquired chemoresistance, and remarkable heterogeneity [5]; recurrence is frequently observed in up to 70 % of cases [6]. In such patients, invasion, metastasis, and chemoresistance may play important roles. Consequently, increasingly sophisticated experiments have been performed to study targeted treatments to improve the 5-year survival rate of patients with ovarian cancer.

MicroRNAs (miRNAs) are a class of small non-coding RNAs that negatively regulate gene expression at post-transcriptional level [7]. It has been demonstrated that a small number of miRNAs actively participate in regulating tumor development; it has been shown that they play different roles in different organs, particularly relating to cancer development [8–12]. It has been determined that miR-23b mediates the various steps in the metastatic process, including tumor growth, invasion, and even angiogenesis by repressing a cohort of prometastatic targets [13]. MiR-23b is downregulated in many cancers and acts as a tumor suppressor [14–16]. From a clinical viewpoint, miR-23b has great potential as a diagnostic and therapeutic agent in some tumors. Our previous study showed that miR-23b was highly expressed in normal ovarian tissues than ovarian carcinoma tissues, and our predicted seed region in the 3′ untranslated regions (3′ UTR) of CCNG1 revealed that it’s a target of miR-23b, thus we investigated the involvement of CCNG1 and miR-23b in ovarian cancer for the first time.

## Methods

### Cell culture

The ovarian cancer cell lines OVCAR3 and HO8910-PM (highly invasive HO8910) were from ATCC. The cisplatin-resistant SKOV3 (SKOV3/DDP) cell line was purchased from the Tumor Cell Bank of the Chinese Academy of Medical Science (Peking, China). The cells were maintained in RPMI 1640 medium supplemented with 10 % fetal bovine serum (FBS), 100 units/mL penicillin and 100 μg/mL streptomycin in a humidified atmosphere of 5 % CO_2_ at 37 °C.

### Proliferation assay

We used 3-(4,5)-dimethylthiazol (−zyl)-3,5-diphenyltetrazolium bromide (MTT; China) to determine the number of viable cells. Briefly, approximately 2.5 × 10^3^ cells/well were seeded in a 96-well plate and allowed to adhere. At different time points, 20 μL MTT solution was added to each well, and the plates were incubated for 4 h. Subsequently, the MTT solution was removed, and 150 μL dimethyl sulfoxide was added and incubated for 10 min before the absorbance was measured at 490 nm.

### Cell cycle analysis

After 48-h incubation, the cells were washed, collected, and fixed in 10 mL ice-cold ethanol (70 %) for 12 h. Then, the cells were washed, incubated with 5 μL RNase (0.25 mg/mL) at 37 °C for 30 min, pelleted, resuspended in 50 μg/mL PI, and incubated for 15 min in the dark at room temperature. Cell cycle analysis was performed by flow cytometric analysis.

### Apoptosis assay

Flow cytometry was performed following PI and FITC-labeled annexin V (KeyGen Biotech, Nanjing, China) staining according to the manufacturer’s protocol. Briefly, after 48-h incubation, cells were washed and resuspended in 200 μL 1× binding buffer at 1 × 10^6^ cells/mL, and incubated with 5 μL FITC–annexin V; after 15-min incubation at room temperature in the dark, 300 μL 1× binding buffer together with 5 μL PI were added to each tube. The samples were incubated for 30 min at room temperature in the dark. Flow cytometry was performed within 1 h.

### Wound healing assay

Cells were seeded at 1 × 10^6^ cells/well in 6-well culture plates. After they had grown to confluence, the monolayer was scratched with a pipette tip (200 μL). The cells were washed with PBS three times and cultured in FBS-free medium, and then photographed at 0, 24, and 48 h. The scratched areas were measured using ImageJ software, after which the wound healing rate was calculated.

### Cell invasion assay

5 × 10^4^ cells were resuspended in FBS-free medium and seeded into the top chambers of Matrigel™-coated Transwell® inserts (BD Bioscience, San Jose, CA, USA). The lower compartments of the chambers contained 10 % v/v FBS as a chemoattractant. After 48-h incubation, the cells on the upper surface of the membrane were wiped away, and the cells on the lower surface of the membrane were washed, fixed, and stained with crystal violet to quantify the extent of invasion.

### Ovarian carcinoma specimens

Specimens from 50 ovarian epithelial carcinomas, 13 benign tumors, 5 borderline tumors, and 6 normal ovarian tissues were collected from patients who underwent surgical resection at the Department of Gynecology of the First Affiliated Hospital of China Medical University (Shenyang, China). Two pathologists confirmed the tumor specimens independently. The China Medical University Ethics Committee approved the study.

### Real-time RT-PCR (real-time RT-PCR)

Real-time RT-PCR was performed from 2 μg total RNA using AMV reverse transcriptase and random primers (Takara). The PCR primers were designed according to the GenBank sequences. The glyceraldehyde-3-phosphate dehydrogenase gene (*GAPDH*) was used as the internal control. The relative gene expression level (the amount of target gene normalized to the endogenous control gene) was calculated using the comparative threshold cycle method: 2^-ΔΔCt^. Hairpin-it™ microRNA and U6 snRNA Normalization RT-PCR Quantitation (GenePharma) were used to examine mature miR-23b.

### Western blotting

Proteins were separated by SDS-PAGE, transferred to polyvinylidene difluoride membranes and immunoblotted overnight at 4 °C with the primary antibodies (1:500), rinsed with TBST, and incubated with 1:5000 secondary antibodies conjugated to horseradish peroxidase (Dako). After applying electrochemiluminescence (ECL) Plus detection reagents (Santa Cruz Biotechnology), protein bands were visualized using X-ray film (Fujifilm, Tokyo, Japan). GAPDH-specific monoclonal antibody (1:2000; Santa Cruz Biotechnology) was used as the internal control.

### In vivo nude mouse tumorigenicity assay

The assay was performed to determine the effects of miR-23b overexpression on the tumorigenicity of OVCAR3 cells in vivo. Mock or hsa-miR-23b–transfected OVCAR3 cells (1 × 10^7^) were injected subcutaneously into the flanks of 4-6-week-old male nude mice (n = 5). Once tumor growth had been established in the mock-treated mice (14 days post-injection), tumor sizes were measured every other day. Tumor volume (mean ± standard deviation) was calculated as length × width^2^/2.

### Immunofluorescence (IF) staining

5-μm frozen section from each sample was fixed in acetone at 4 °C overnight. After washing, sections were blocked with 1 % bovine serum albumin for 30 min and incubated overnight at 4 °C with rabbit anti-human CCNG1 antibody (1:50). Then sections were incubated with anti-rabbit IgG–FITC (1:100, Santa Cruz Biotechnology) for 2 h at room temperature in the dark. Nuclei were stained with diaminophenylindole (1 μg/mL; Sigma-Aldrich) for 5 min at room temperature. Coverslips were mounted and imaged using a laser confocal microscope (Olympus, Tokyo, Japan).

### Dual-luciferase reporter assay

A293 cells were first seeded at 60–80 % confluence in 24-well plates for 12 h. For the dual-luciferase reporter assays, cells were transiently transfected with Wt or mutated reporter plasmid and miR-23b or control-miR. Firefly luciferase activity was measured using a Dual-Luciferase Assay (Promega) 48 h after transfection, and the results were normalized with *Renilla* luciferase. Each reporter plasmid was transfected at least three times, and each sample was assayed in triplicate.

### Statistical analysis

Statistical evaluation was performed using Spearman’s rank correlation coefficient to analyze ranked data; the Mann–Whitney *U* test was used to differentiate the means of different groups. A *p*-value of < 0.05 was considered statistically significant. SPSS v. 17.0 (SPSS) was used to analyze all data.

## Results

### Correlation of *MIR23B* and cyclin G1 (*CCNG1*) mRNA expression with pathogenesis and aggressiveness of ovarian carcinoma

**Fig. 1:**
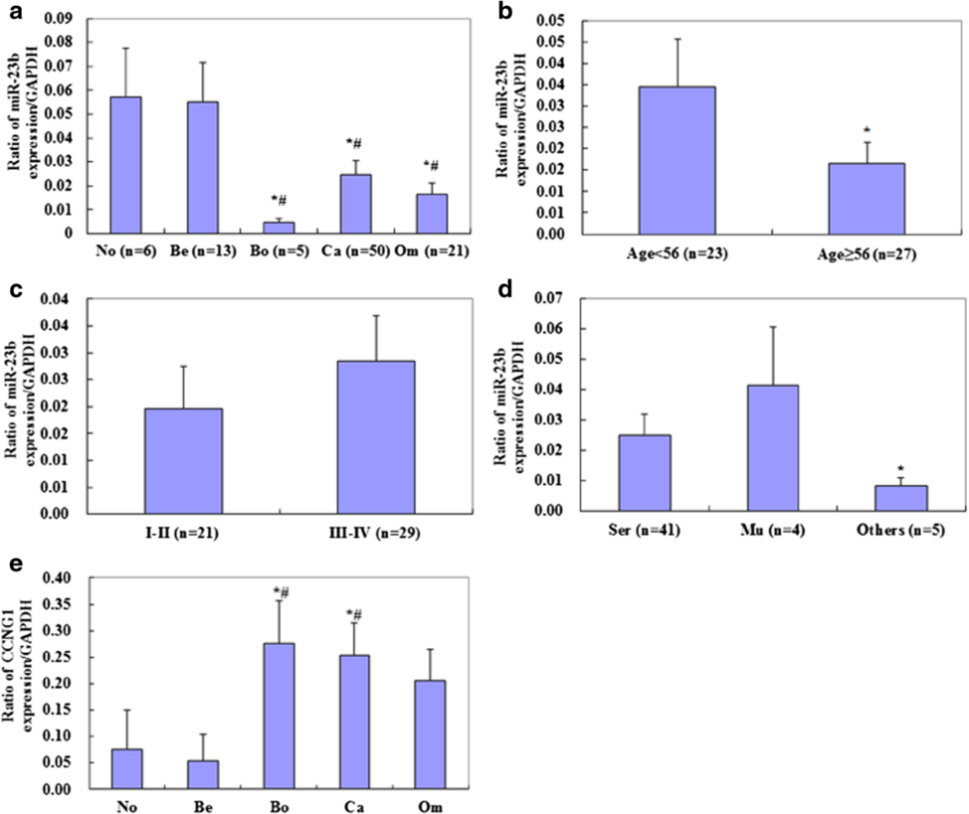
Correlation of *MIR23B* and *CCNG1* mRNA expression with pathogenesis and aggressiveness of ovarian carcinoma. **a** *MIR23B* mRNA expression was significantly lower in the ovarian carcinomas and borderline ovarian tumors than in the normal ovarian tissues and benign tumors; **b** there were significant differences in expression among age and **d** pathological subtype (mucinous vs. other types) in the ovarian carcinoma tissues. **c** There were no significant differences among FIGO stage (I/II vs. III/IV) in ovarian carcinoma. **e** *CCNG1* mRNA expression was significantly lower in the normal ovarian tissues and benign ovarian tumors than in the ovarian carcinomas. *p < 0.05 vs. normal ovarian tissues; ^#^ *p* < 0.05 vs. benign ovarian tumors. No = Normal ovarian tissues, Be = benign ovarian tumors, Bo = borderline ovarian tumors, Om = omentum tumors, Mu = mucinous carcinoma, Ser = serous carcinoma

We quantified *MIR23B* and *CCNG1* mRNA expression in normal ovary tissue, benign and borderline tumors, and primary ovarian carcinoma using real-time PCR. *MIR23B* mRNA expression was significantly lower in the ovarian carcinomas and borderline tumors than in the normal ovarian tissues and benign tumors (Fig. 1a, *p* < 0.05), and there were significant differences in expression among ages (Fig. 1b, *p* < 0.05) and pathological subtypes (mucinous vs. other types, Fig. 1d, *p* < 0.05). However, there were no significant differences among International Federation of Gynecology and Obstetrics (FIGO) stages (I/II vs. III/IV, Fig. 1c, *p* > 0.05) and differentiation (well vs. poor and moderate, data not shown, *p* > 0.05) in ovarian carcinoma. *CCNG1* mRNA expression was significantly lower in the normal ovarian tissues and benign ovarian tumors than in the ovarian carcinomas (Fig. 1e, *p* < 0.05).

### Effects of miR-23b transfection on ovarian carcinoma cell phenotype in vitro

**Fig. 2:**
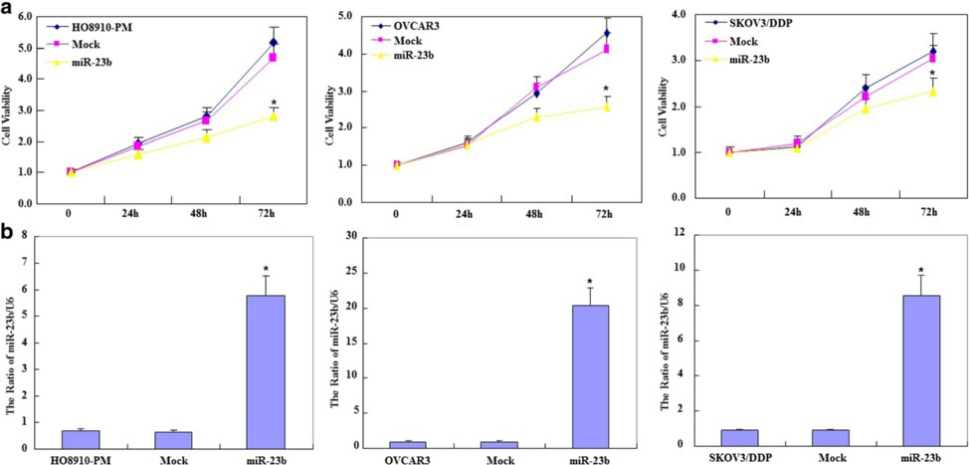
Effects of miR-23b transfection on ovarian carcinoma cell proliferation in vitro. **a** Following miR-23b transfection, OVCAR3, HO8910-PM, SKOV3/DDP cell lines exhibited significantly slower growth and **b** higher levels of mature miR-23b expression than the control and mock-transfected cells. Results are representative of three separate experiments; data are expressed as the mean ± standard deviation, * *p* < 0.05

**Fig. 3:**
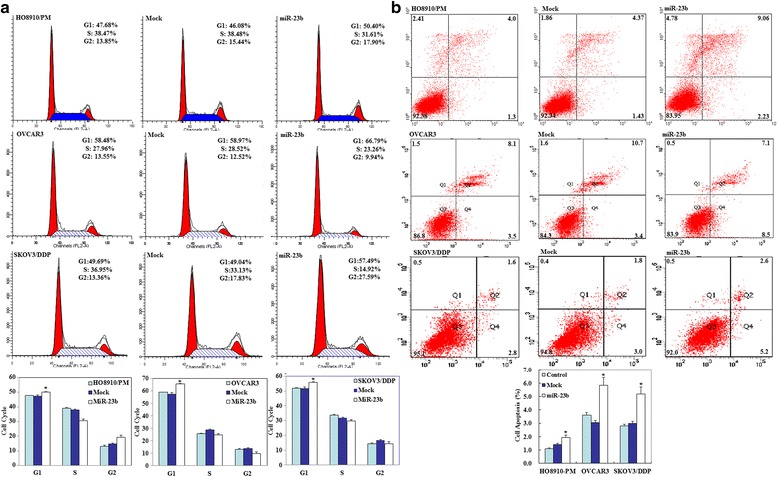
Effects of miR-23b transfection on ovarian carcinoma cell cycle and apoptosis in vitro. MiR-23b transfection induced **a** G_1_ arrest and **b** early apoptosis in OVCAR3, HO8910-PM, and SKOV3/DDP cells as compared to the control and mock-transfected cells. Results are representative of three separate experiments; data are expressed as the mean ± standard deviation, * *p* < 0.05

**Fig. 4:**
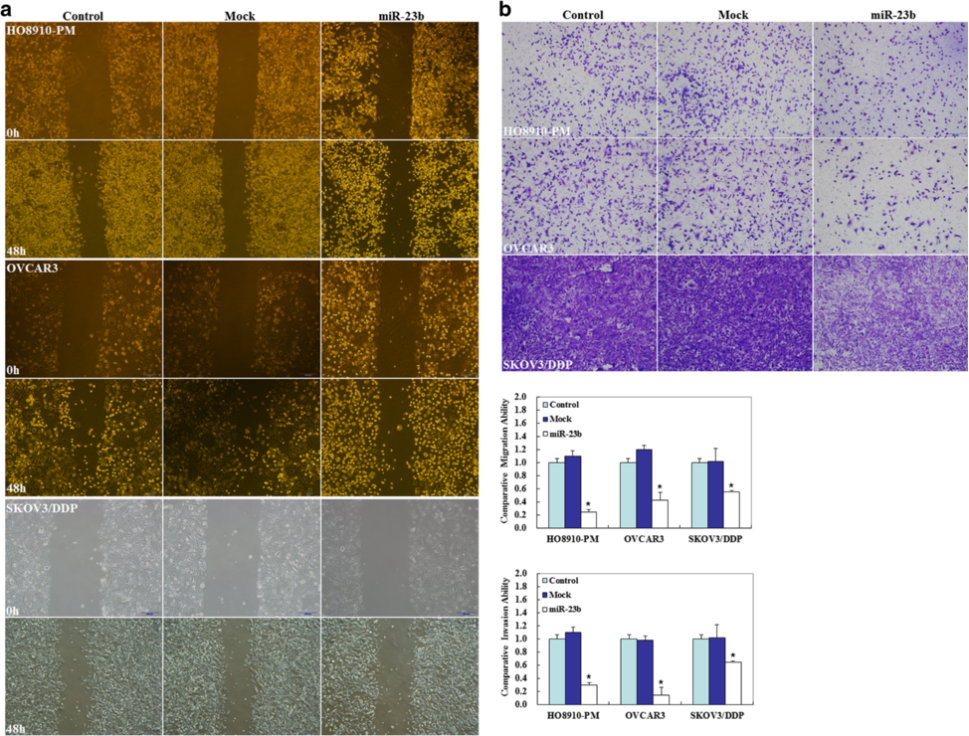
Effects of miR-23b transfection on the invasive and metastatic ability of ovarian carcinoma cell lines. After transfection with the miR-23b mimic, ovarian carcinoma cells showed **a** lower migration and **b** slower invasion as compared with the control and mock-transfected cells. Results are representative of three separate experiments; data are expressed as the mean ± standard deviation, *p < 0.05

We transfected OVCAR3, HO8910-PM, SKOV3/DDP cells with miR-23b. The transfected cells exhibited significantly slower growth (Fig. 2a, *p* < 0.05) but higher levels of mature miR-23b expression than the control and mock-transfected cells (Fig. 2b, *p* < 0.05). MiR-23b overexpression significant induced G_1_ arrest (Fig. 3a, *p* < 0.05), higher levels of apoptosis (Fig. 3b, *p* < 0.05) and reduced cell migration (Fig. 4a, *p* < 0.05) and invasion (Fig. 4b, *p* < 0.05) compared to the control and mock-transfected cells.

### Effects of miR-23b transfection on ovarian carcinoma cell genotype in vitro

**Fig. 5:**
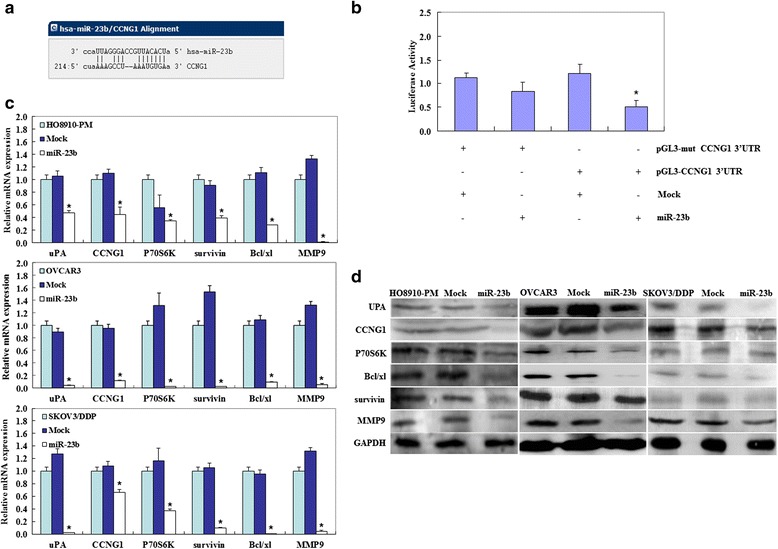
Effects of miR-23b transfection on ovarian carcinoma cell genotype in vitro. **a** The predicted seed region in the 3′ UTR of *CCNG1* revealed that *CCNG1* was direct target of miR-23b, as predicted by microRNA.org; **b** dual-luciferase reporter assay indicated that miR-23b significantly decreased the relative luciferase activity of the wild-type *CCNG1* 3′ UTR as compared with the mutant *CCNG1* 3′ UTR, indicating that miR-23b may directly bind to the 3′ UTR of *CCNG1*. **c **& **d** MiR-23b overexpression reduced CCNG1, uPA, P70S6K, Bcl-xL, survivin and MMP9 mRNA or protein expression. * *p* < 0.05

The predicted seed region in the 3′ untranslated regions (3′ UTR) of *CCNG1* revealed that it is direct target of miR-23b (Fig. 5a); dual-luciferase reporter assay indicated that miR-23b significantly decreased the relative luciferase activity of the wild-type *CCNG1* 3′ UTR as compared with the mutant *CCNG1* 3′ UTR, indicating that miR-23b may directly bind to the 3′ UTR of *CCNG1* (Fig. 5b). Reverse transcription (RT)-PCR (Fig. 5c, *p* < 0.05) and western blotting (Fig. 5d) showed that miR-23b overexpression reduced CCNG1, urokinase (uPA), P70S6K, Bcl-xL, survivin, and matrix metallopeptidase 9 (MMP9) mRNA or protein expression.

### MiR-23b inhibited tumor growth in vivo

**Fig. 6:**
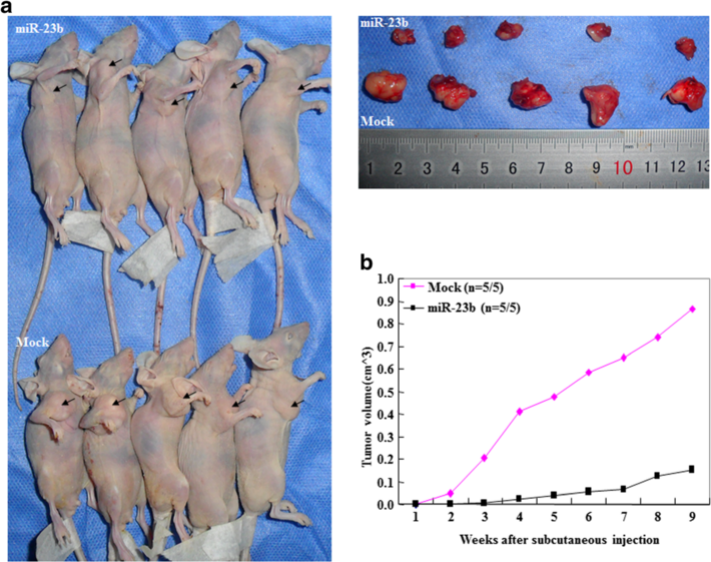
MiR-23b inhibited tumor growth in vivo. **a** Tumor xenograft volume in nude mice treated with hsa-miR-23b was smaller than that in mock-treated mice. **b** Tumor xenograft growth in miR-23b–treated nude mice was slower than that in the mock group from day 4 and week 2 onwards

The tumor xenograft volume in nude mice treated with miR-23b was smaller than that in the mock-treated mice (Fig. 6a, *p* < 0.05). From day 4 and week 2 onwards, the tumor xenograft growth in the miR-23b–treated BALB/c mice was slower than that in the mock group (Fig. 6b, *p*_day 4_ < 0.05; p_deviation of tumor xenograft volume [DV]_ < 0.01, and *p*_week 2_ < 0.05; *p*_DV_ < 0.01), and the DV increased in the latter period.

### MiR-23b downregulated CCNG1 expression in tumor xenografts in vivo

**Fig. 7:**
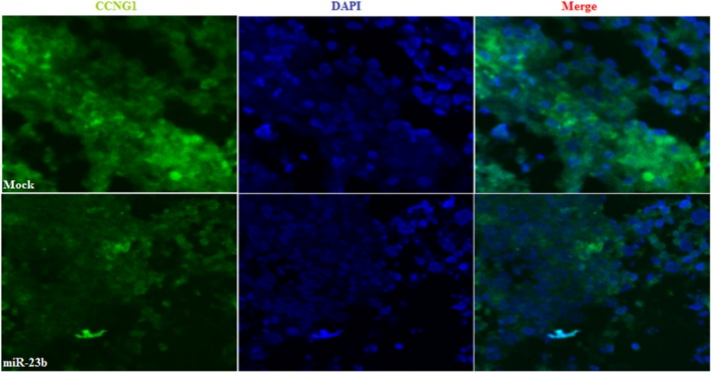
MiR-23b downregulated CCNG1 expression in tumor xenografts in vivo. IF experiments indicated that CCNG1 expression in the tumor xenografts of miR-23b–treated nude mice were decreased compared with that in the mock-treated nude mice

Immunofluorescence staining (IF) indicated decreased CCNG1 expression in the tumor xenografts of the miR-23b–treated nude mice compared with that in the mock-treated nude mice (Fig. 7).

## Discussion

MiRNAs regulate their target genes by affecting base pairing to their 3′ UTRs, resulting in mRNA degradation or inhibition of translation [11]. An increasing number of studies have revealed that miRNAs are promising diagnostic and prognostic molecular biomarkers as well as therapeutic targets in cancer [17, 18]. Series of studies has reported that miR-23b acts as a tumor suppressor in different cancers [19]. Majid et al. showed that miR-23b has diagnostic/prognostic significance and directly targets the oncogenic ZEB1 in bladder cancer [14]. He et al. reported that miR-23b expression levels in prostate carcinoma (PCa) tissues was significantly correlated with that of peroxiredoxin 3 (*PRDX3*) and that miR-23b may be involved in the response of PCa cells to hypoxic stress, therefore gene therapy using miRNA mimics may be useful as PCa therapy [20]. Our results show that *MIR23B* mRNA expression was significantly lower in ovarian carcinomas and borderline tumors than in normal ovarian tissues and benign tumors, and the expression among age and pathological subtypes (mucinous vs. other types) was significantly different. These findings indicate that miR-23b might affect ovarian epithelial carcinogenesis and the subsequent progression. Therefore, we explored the function and molecular mechanism of miR-23b in ovarian cancer cell lines.

Ovarian cancer cells transfected with miR-23b had significantly slower growth than the negative control– and mock-transfected cells, and there was significantly induced G_1_ arrest and apoptosis and reduced cell invasion and migration, suggesting miR-23b may inhibit ovarian carcinoma tumorigenesis and progression. Moreover, the predicted seed region showed that miR-23b targets CCNG1 3′ UTR, which was convinced by the dual-luciferase reporter assay. We also found that miR-23b transfection decreased CCNG1 mRNA and protein expression. CCNG1 was first identified as a p53-regulated transcript induced by DNA damage. It has been proposed that these events underpin CCNG1 participation in the enforcement of the p53-dependent G_1_–S and G_2_ checkpoints responsive to DNA damage [21]. Some have suggested that CCNG1 might function as an oncogenic protein [22, 23] and play a pivotal role in the initiation and metastasis of hepatocellular carcinoma [24]. Russell et al. reported that CCNG1 amplification is associated with significantly shorter postsurgical survival in patients with ovarian cancer who have received adjuvant chemotherapy with taxanes and platinum compounds [21]. These results suggest that miR-23b may inhibit ovarian cancer tumorigenesis and progression by targeting CCNG1.

In this study, we also found that miR-23b overexpression downregulated uPA expression, which is in line with the findings of Salvi et al. [15], who reported that miR-23b overexpression leads to uPA downregulation and decreased migration and proliferation ability in hepatocellular carcinoma cells. Furthermore, miR-23b overexpression also downregulated the expression of P70S6K, survivin, Bcl-xL, and MMP9 mRNA and protein. It has been established that uPA is integral to cell differentiation, migration, tissue remodeling under physiological and pathological conditions, and may be a potential diagnostic biomarker and therapeutic target in cancer. Significant elevation of uPA protein levels in primary ovarian cancer tissue has been associated with poor prognosis and disease progression [25–29]. The uPA system plays an important role in many pathophysiological processes, such as cell differentiation, migration, tissue reconstruction, and matrix dissolution [30]. The aberrant expression of phosphorylated P70S6K might contribute to the pathogenesis, growth, invasion, and metastasis of cancer [31]. Wang et al. reported that uPA promoted carcinoma cell proliferation by stimulating P70S6K activation [32]. Survivin, a member of the inhibitors of apoptosis protein family, is expressed during development and in various human cancers. Lee et al. reported that downregulating survivin suppressed uPA through the transcription factor JunB [33]. Ryan et al. reported that survivin expression in breast cancer predicts clinical outcome and is associated with uPA [34]. Furthermore, Zhou et al. reported that Bcl-xL overexpression strongly enhanced uPA in pancreatic ductal adenocarcinoma cells [35]. Therefore, we suggest that miR-23b reduces cell proliferation by downregulating CCNG1 and uPA/P70S6K expression, suppresses cell migration and invasion by downregulating uPA/MMP9 expression, and induces apoptosis by downregulating survivin and Bcl-xL/uPA expression.

We found that *CCNG1* mRNA expression was significantly lower in the normal ovarian tissues and benign ovarian tumors than in the borderline ovarian tumors and ovarian carcinomas. Our subsequent in vivo tumor xenograft studies showed that miR-23b inhibited tumor growth and decreased CCNG1 expression. Recent studies have reported that *CCNG1* gene therapy has been developed and has undergone phase I/II clinical trials for treating colorectal and pancreatic cancer [36]. We suggest that miR-23b may also be a potential suppressor of ovarian carcinoma by targeting *CCNG1*.

Our findings show that miR-23b may inhibit ovarian cancer tumorigenesis and progression by downregulating CCNG1 and the expression of the relevant genes, and that miR-23b is a potentially novel application for regulating ovarian carcinoma progression.

## Conclusion

This is the first demonstration that miR-23b may inhibit EOC tumorigenesis and progression by targeting CCNG1 and modulating the expression of the relevant genes. These findings indicate that miR-23b is a potential suppressor of ovarian carcinoma tumorigenesis and progression. The involvement of miR-23b–mediated CCNG1 downregulation in inhibiting EOC aggressiveness may yield further insight into the molecular mechanisms underlying cancer aggressiveness.

## Abbreviations

CCNG1: cyclin G1
EOC: epithelial ovarian carcinoma
FIGO stages: International Federation of Gynecology and Obstetrics stages
GAPDH: glyceraldehyde-3-phosphate dehydrogenase gene
IF: immunofluorescent staining
miR-23b: microRNA 23b
MMP9: matrix metallopeptidase-9
MTT: 3-(4,5)-dimethylthiazol (−zyl)-3,5-diphenyltetrazolium bromide
RT-PCR: real-time RT-PCR
SDS-PAGE: sodium dodecyl sulfate–polyacrylamide gel electrophoresis
TBST: tris-buffered saline with tween 20®
